# The Venereal Clinics of the Seamen's Hospital Society

**Published:** 1917-06-09

**Authors:** 


### THE VENEREAL CLINICS OF THE SEAMEN'S HOSPITAL SOCIETY.

Venereal clinics working in accordance with the Local Government Board's instructions have been established at the main hospital, Greenwich, and also at the branch hospital at the Albert Docks.

*The Dreadnought Hospital, Greenwich.*—The venereal clinic began its operations here on January 1, 1917. Although the majority of the male patients are sailors, about 25 per cent. come from all over London and the country outlying it.

The hospital has devoted for the purposes of the clinic the necessary accommodation on the ground floor, and also the use of the hospital's pathological laboratory and its dispensary.

Out-patients arriving in the hospital are received in the general waiting-room and thence conducted to a special waiting-room adjoining the inspection-room, where the medical officer in charge of the clinic, his clerk, and head dresser are in attendance. The patients are then examined by the medical officer in charge, who immediately makes out a history card bearing their identification number, after which they receive a red card also bearing their identification number, upon which is entered the date for their next attendance. Their names and addresses are then entered in the private register opposite to their identification number.

Some patients are then prescribed for at once; others are sent to the pathological laboratory for the blood test, the result of which is delivered to the medical officer in charge of the clinic before the patient's next attendance.

After all the waiting patients have been interviewed, those to receive injections of salvarsan or one of its substitutes pass one by one to another room, where they are duly injected, and, having been advised when to return, are taken to a rest-room where they lie down for one hour at least before leaving the hospital.

All patients are told of the effects of syphilis if it is allowed to run its course, and the great hope of a permanent cure if they carry out the instructions given them for the two years following on the third injection. They then receive the pamphlets issued by the Local Government Board relative to the disease.

The medical officer finds expression of gratitude on the part of nearly all the patients and an evident resolve to carry out the instructions given them. How far they will carry out their resolve, of course only the future can tell.

The subsidiary books in use for the purpose of keeping constantly up to date the statistics required by the Local Government Board are as follows:—

1. Diary for entry of patients' numbers and their date to attend.
2. Attendance record of practitioners.
3. Injection record book.
4. Dressers' book for recording irrigations, etc.
5. The clinic record made up after each clinic from the foregoing subsidiary and the main books.

The clinic is undoubtedly a success, and should the number of new patients keep up to the present average with the inevitable large increase of attendances, it will be necessary in the near future that the Society should find more room for the patients and also arrange for longer hours for the clinics, or perhaps hold clinics five days a week instead of the present three.

As the reports of the ways and work of the clinic are circulated by word of mouth from those who have been patients, it is probable that their accounts of the sympathetic manner in which they are cared for and the privacy of the whole arrangements, in addition to the results of the treatment, will rapidly increase the monthly number of new patients. During the month of March (1917) 119 new patients attended the clinic, on 570 occasions. The attendances for all purposes for the quarter were 1,289, while the in-patient days of treatment were 2,565.

The clinic at the Albert Dock Hospital was opened on April 2 last. All arrangements have been made for the clinic on precisely the same lines as those existing at the Dreadnought Hospital, Greenwich, and the records and subsidiary books kept are replicas of those at Greenwich.

It is very satisfactory that at Greenwich as elsewhere the venereal clinics have proved so successful, and it is to be hoped that the experience gained during this first year of trial will enable the work to be carried on with even greater success in subsequent years. Already most of these clinics have been at work for nearly six months, and the time is approaching when arrangements must be made for the extension of these clinics next year.

